# Compiled data set of exact NOE distance limits, residual dipolar couplings and scalar couplings for the protein GB3

**DOI:** 10.1016/j.dib.2015.08.020

**Published:** 2015-09-04

**Authors:** Beat Vögeli, Simon Olsson, Roland Riek, Peter Güntert

**Affiliations:** a Laboratory of Physical Chemistry, Swiss Federal Institute of Technology, Vladimir-Prelog-Weg 2, ETH-Hönggerberg, CH-8093 Zürich, Switzerland; b Institute for Research in Biomedicine, Via Vincenzo Vela 6, CH-6500 Bellinzona, Switzerland; c Institute of Biophysical Chemistry, Center for Biomolecular Magnetic Resonance, and Frankfurt Institute for Advanced Studies, J.W. Goethe-Universität, Max-von-Laue-Str. 9, 60438 Frankfurt am Main, Germany; d Graduate School of Science, Tokyo Metropolitan University, Hachioji, Tokyo 192-0397, Japan

## Abstract

We compiled an NMR data set consisting of exact nuclear Overhauser enhancement (eNOE) distance limits, residual dipolar couplings (RDCs) and scalar (*J*) couplings for GB3, which forms one of the largest and most diverse data set for structural characterization of a protein to date. All data have small experimental errors, which are carefully estimated. We use the data in the research article Vogeli et al., 2015, Complementarity and congruence between exact NOEs and traditional NMR probes for spatial decoding of protein dynamics, J. Struct. Biol., 191, 3, 306–317, doi:10.1016/j.jsb.2015.07.008 [1] for cross-validation in multiple-state structural ensemble calculation. We advocate this set to be an ideal test case for molecular dynamics simulations and structure calculations.

Specifications table *[please fill in right-hand column of the table below]*

**Table:** 

Subject area	*Physical chemistry, structural biology*
More specific subject area	*Nuclear magnetic resonance, NMR*
Type of data	*Exact NOE distance restraints, J couplings, RDCs files, table, figure*
How data was acquired	*Solution NMR*
Data format	*CYANA input files*
Experimental factors	*–*
Experimental features	*All data has been acquired at 298* *K; typically, the GB3 NMR samples contained 350–500* *µl of 2–4* *mM protein solution in 97%/3% or 95%/5% H*_*2*_*O/D*_*2*_*O, 50* *mM potassium phosphate buffer, pH 6.5–7.0, and 0.5* *mg/ml sodium azide*
Data source location	*Zurich, Switzerland; Bethesda, MD, USA*
Data accessibility	*data is with this article*

Value of the data *[describe in 3–5 bulleted points why this data is of value to the scientific community]*

• one of the largest and most diverse NMR data sets for characterizing the structure and dynamics of a protein to date
• extensive error analysis guarantees high reliability of the data set
• ideal for validation of structure calculation programs and molecular dynamics simulations

## 1 Data

We compiled 923 exact nuclear eNOEs distance limits (upper distance limits in CYANA format in file ‘GB3.upl’; lower distance limits in CYANA format in file ‘GB3.lol’), 61 conventional NOEs for aromatics (upper distance limits in CYANA format in file ‘GB3.upl’), 1477 RDCs (file ‘GB3.rdc’), 225 ^3^*J* scalar couplings (‘GB3.cco’), and 52 torsion angle restraints (‘GB3.aco’) for the third immunoglobulin binding domain of protein G (GB3) (Table 1) [1]. To that purpose, the previously published set consisting of 884 eNOEs [2,3] was supplemented by eNOEs involving methylenes with a degenerate proton pair and Val with a degenerate pair of methyl groups. The RDCs were collected from the literature, in total originating from 8 different alignment conditions. 1335 RDCs were obtained from spin pairs located in the backbone, 129 in side chains, and 13 bridging the backbone and side chains. All *J* couplings, most of which are taken from the literature, extend over three covalent bonds, of which 147 are from the backbone, and 78 between a backbone and a side-chain spin. In the distance limit, RDC and *J* coupling CYANA input files, the first six columns describe the two involved atoms. For the distance restraints, the seventh column is the upper or lower distance restraint. In the RDC file, the seventh to eleventh columns contain the measured value, the experimental error, the relative weight (always 1), the alignment tensor number and a constant collecting physical constants of the specific coupling. The magnitudes and rhombicities of the alignment tensors are indicated in the file header. In the *J* coupling file, the seventh to twelfth columns contain the measured value, the experimental error, the relative weight (always 1), and the coefficients C, B and A of the Karplus equations. In the torsion angle restraint CYANA input file, the first three columns identify the torsion angle, and the fourth and fifth columns list the lower and upper bounds. We also added 41 intraresidual and 46 sequential *D*_HNHα_ RDCs, which cannot be used in CYANA structure calculations due to the flexible distances between the nuclei (files ‘GB3_rdc_3Dhnha.f’ and ‘GB3_rdc_4Dhnha.f’, respectively).

## 2 Experimental design, materials and methods

### 2.1 eNOEs

All cross-relaxation rate constants presented in references [2,3] were taken. Upper and lower limits of the distance restraints were set with an allowed distance range of 0% and +15 and −15% for bidirectional and unidirectional eNOEs, respectively. However, NOEs involving methyl groups were processed differently. In this study, the CYANA protocol was executed with an individual treatment of each methyl proton by *r*^−6^ summation of the corresponding distances. Therefore, previous input distance restraints were scaled by a factor of 3^−1/6^=0.83268 per methyl group such that the corresponding cross-relaxation rate constant is a sum over all individual contributions. This would be strictly true if the methyl motion was slow (slower than nanoseconds). Since there is fast rotation present as well, we added an additional tolerance of ±8.5%.

eNOEs that involve either methylene groups with degenerate chemical shifts or chemically equivalent methyl groups in Val and Leu were added to the previous data set. The apparent cross-relaxation rate constants were fitted to the same formulae as used for single atoms or methyl groups, corrected for spin diffusion [4] and normalized to the equivalent of a superposition of contributions from all pairs of single atoms. Note that this is an approximation because the spins do not undergo fast exchange. Instead, the spectral peaks are superpositions of the individual buildup/decay curves. Therefore, all upper limits and lower limits were given an additional tolerance of 5% in addition to the 0/15% for bidirectional/unidirectional eNOEs.

### 2.2 Residual dipolar couplings

^1^*D*_HN,N_ (43 Hz range) and ^1^*D*_Cα,Hα_ (92 Hz range) RDCs of wild-type GB3 under alignment induced by Pf1 filamentous phages (tensor 1) were taken from reference [5]. For ^1^*D*_HN,N_, the errors were uniformly set to 0.5 Hz because half the pairwise r.m.s. deviation from values obtained from a new sample was 0.57 Hz, and singular value decomposition (SVD) fits of the RDCs to a 160-member multi-state ensemble [6], the RDC-refined X-ray structure (PDB 2OED) [7], and RDC-proton optimized 2OED [8–10] yield fitting errors of 0.54 Hz, 0.76 Hz, and 1.29 Hz, respectively. Analogously, the errors for ^1^*D*_Cα,Hα_ were uniformly set to 1.5 Hz because SVD fits to the same structures yield fitting errors of 1.67 Hz, 2.02 Hz, and 2.51 Hz, respectively, and if the alignment tensor is taken from the H^N^–N RDCs, only slightly larger errors are observed with 2.07 Hz, 2.40 Hz, and 3.52 Hz. An initial tensor estimate was obtained from SVD fit of the H^N^–N RDCs to the RDC-refined X-ray structure [8–10] and omission of the highly mobile residues 12, 40, and 41. This tensor was then used for both ^1^*D*_HN,N_ and ^1^*D*_Cα,Hα_. The relative scaling is –2.0327 (corresponding to the bond lengths of 1.02 Å and 1.09 Å).

For the deuterated mutants K19AD47K (tensor 2), K19ED40N (tensor 3), K19EK4A-C-His6 (tensor 4), K19EK4A-N-His6 (tensor 5), and K19AT11K (tensor 6) two slightly different experiments were run to obtain H^N^–N and C^α^–C’ RDCs under alignment via Pf1 [10]. For these data sets, the averages are used and the errors are half the individual pairwise differences. For the 54, 54, 52, 54, 54 ^1^*D*_HN,N_ values, the overall r.m.s. deviations for the sets corresponding to tensors 2, 3, 4, 5, 6 are 0.14, 0.17, 0.16, 0.11, 0.07 Hz for ranges of 28, 30, 35, 27, 30 Hz, respectively. If only one value was available, the error was set to twice the overall r.m.s. deviation. For the 54, 54, 50, 54, 54 ^1^*D*_Cα,C’_ values the errors for the sets corresponding to tensors 2, 3, 4, 5, 6 are 0.041, 0.078, 0.152, 0.073, 0.047 Hz for ranges of 6.2, 6.2, 7.4, 6.7, 7.3 Hz, respectively. If only one value was available, the error was set to twice the overall r.m.s. deviation. The scaling of ^1^*D*_Cα,C’_ relative to ^1^*D*_HN,N_ is −0.1866 (corresponding to the bond lengths of 1.525 Å and 1.02 Å). For a seventh alignment condition under Pf1 (tensor 7, mutant K19EK4A), only one data set of ^1^*D*_HN,N_ (37 Hz range) and ^1^*D*_Cα,C’_ (8.1 Hz range) is available. The error was set uniformly to twice the largest error of the other five data sets (0.34 Hz and 0.30 Hz).

For H^N^–C’, one set of RDCs is available for each mutant yielding tensors 2–6 [10]. The relative scaling of the coupling is −0.3123, assuming an interatomic distance of 2.035 Å corresponding to lengths of 1.020 Å and 1.329 Å for the H^N^–N and C’–N bonds and a bond angle of 119.5° [11]. R.m.s. deviations from back-calculated values obtained from SVD are typically 0.4 Hz (using a directly fitted tensor or a tensor obtained from ^1^*D*_HN,N_). Therefore, the errors were uniformly set to 0.2 Hz.

For each of the protonated mutants K19AD47K (tensor 2), K19ED40N (tensor 3), K19EK4A-C-His6 (tensor 4), K19EK4A-N-His6 (tensor 5), K19AT11K (tensor 6), and K19EK4A (tensor 7), one ^1^*D*_Cα,Hα_ data set is available [9]. To account for Pf1 concentration differences, the values were rescaled by the slopes between the ^1^*D*_H,N_ values obtained from the protonated samples (not used in this study) and the deuterated samples (see above). The alignment tensors are assumed to be the same as those determined from the ^1^*D*_HN,N_ sets of the deuterated samples. R.m.s. deviations from back-calculated values obtained by SVD are typically 1.5 Hz (or ca. 2.0–3.5 Hz using the tensors obtained from ^1^*D*_HN,N_). Therefore, the errors are uniformly set to 1.0 Hz.

For the structure calculations, tensors 2–7 were determined from SVD with all measured RDCs in the backbone.

^2^*D*_Cβ,Hα_, ^1^*D*_Cβ,Hβ2_, ^1^*D*_Cβ,Hβ3_ and ^1^*D*_Hβ2,Hβ3_ values were obtained from alignment with Pf1 phage [12]. A 3D HBCBCA type experiment provided four independent values for ^2^*D*_Cβ,Hα_, which allows for an estimation of individual errors. ^1^*D*_Cβ,Hβ2_, ^1^*D*_Cβ,Hβ3_ and ^1^*D*_Hβ2,Hβ3_ values are obtained from linear combinations of the effectively measured ^1^*D*_Cβ,Hβ2_–^1^*D*_Hβ2,Hβ3_, ^1^*D*_Cβ,Hβ3_–^1^*D*_Hβ2,Hβ3_, and ^1^*D*_Cβ,Hβ2_+^1^*D*_Cβ,Hβ3_ values. Here, the errors of these values were propagated into individual errors of the couplings of interest. Sample conditions were similar to those of the previously mentioned measurement of ^1^*D*_HN,N_ with Pf1 yielding tensor 1. ^1^*D*_Cα,Hα_ couplings obtained from the same experiment were compared (Pearson’s correlation coefficient 0.99) and rescaled to the ^1^*D*_Cα,Hα_ couplings mentioned above in order to estimate the alignment tensor magnitude relative to tensor 1. Then, tensor 1 was used for the structure calculations (the scaling factor was 0.814 and the errors were not scaled).

^1^*D*_Cβ,Hβ_ (Val, Ile, Thr), ^1^*D*_Cβ,H3β_ (Ala), ^1^*D*_Cγ1/2,H3γ1/2_ (Val, Ile, Thr) and ^1^*D*_Cδ1/2,H3δ1/2_ (Leu, Ile) values were obtained from alignments with Pf1 phage and PEG [6]. For Pf1, a set of ^1^*D*_Cα,Hα_ recorded on the same sample was used to scale the couplings such that the previously mentioned tensor 1 for alignment with Pf1 could be used (scaling factor 0.90 to account for Pf1 concentration difference, Pearson’s correlation coefficient 0.99) and also for the estimate of the error (r.m.s. deviation between those two sets is 2.32 Hz, which gives an error of ca. 2.32/2^1/2^ Hz=1.5 Hz here). The couplings within methyl groups were scaled by −1/3.17 such that they could be used as effective ^1^*D*_Cα,Cβ_, ^1^*D*_Cβ,Cγ1/2_ and ^1^*D*_Cγ(1),Cδ1/2_ couplings in the structure calculation [13]. The rescaled errors would be ca. 0.2 Hz, but were uniformly set to 0.5 Hz. For PEG, no ^1^*D*_HN,N_ couplings were available and the alignment tensor was determined from a set of ^1^*D*_Cα,Hα_ obtained in the same experiment (tensor 8). The measurements were carried out once. The errors were estimated as follows: An SVD from the ^1^*D*_Cα,Hα_ couplings of Pf1 gave an r.m.s. deviation of ca. 2.7 Hz (this tensor was not used in the structure calculations). An analogous SVD from the ^1^*D*_Cα,Hα_ couplings of PEG yielded an r.m.s. deviation of ca. 1 Hz, while the coupling amplitude is half as large. Thus, the RDC data from PEG appear 1.5 times better and if it is assumed that the absolute measuring errors are similar for PF1 and PEG, it would be safe to use the same errors for PEG as for Pf1. However, it is likely that tensor 8 obtained from ^1^*D*_Cα,Hα_ is not as accurate as tensor 1, which is obtained from ^1^*D*_HN,N_. Therefore, a uniform error of 2 Hz was chosen for ^1^*D*_Cα,Hα_ and ^1^*D*_Cβ,Hβ_. The errors of the couplings involving methyl groups were uniformly set to 0.5 Hz.

Next, all fitted alignment tensors were corrected for the rescaling due to uniform motion throughout the molecule. Iterative re-determination of the tensors increases them by 4% in the first cycle of a two-state ensemble calculation (CYANA target function value changes by +0.91 Å^2^, or −1.08 Å^2^ when using effective bond lengths) and converges to an increase of about 5% after the second cycle. This result is in good agreement with a tensor rescaling by 1/0.95 based on SVD of the 160-member ensemble calculated by Schwieters and Clore [6]. Here, we use the re-determined tensor after the initial two-state ensemble calculation.

In addition, intraresidual ^3^*D*_HN,Hα_ and sequential ^4^*D*_HN,Hα_ RDCs taken from Ref. [5] were used for validation only.

### 2.3 Scalar couplings

^3^*J*_HN,Hα_ values are averages over couplings derived from CT-MQ(^1^H^N^,^13^C^α^)+SQ(^1^H^N^)-HNCA spectra and *J*-modulated HMQC spectra [14,15]. Based on the pairwise r.m.s. deviation between the values obtained from the two types of measurements, the error of their averaged values equals 0.14 Hz [14]. For each coupling, we use the averages over two sets of each type as CYANA input and the standard deviation as input error (overall 0.15 Hz). If both data sets of one type (in our case only *J*-modulated HMQC spectra) are missing the error was set to 0.3 Hz. The averaged measured values were corrected for the residual dipolar couplings between H^N^ and H^α^ due to the natural alignment of GB3 in the magnetic field at 600 MHz. The alignment tensor was estimated from sums of H^N^–N residual dipolar and scalar couplings at 500 and 800 MHz fields. The H^N^–H^α^ RDCs were back-predicted from an RDC-refined X-ray structure [8–10].

^3^*J*_HN,Cβ_ values are averages over couplings derived from a CT-MQ(^1^H^N^,^13^C^α^)-HNCA and a HNCA[CB] E.COSY experiment [14]. The individual errors were based on the pairwise r.m.s. deviation between these two sets of values, with an overall error of their averaged values of 0.07 Hz [14]. If the value of one data set was missing, 0.1 Hz was used.

^3^*J*_HN,C′_ values are averages over couplings derived from a CT-MQ(^1^H^N^,^13^C^α^)-HNCA and a HNCA[C′] E.COSY experiment [14]. The individual errors were based on the pairwise r.m.s. deviation between these two sets of values, with an overall error of their averaged values of 0.1 Hz [14]. If the value of one data set was missing, 0.2 Hz was used.

The Karplus curve coefficients for ^3^*J*_HN,Hα_, ^3^*J*_HN,Cβ_ and ^3^*J*_HN,C′_ were determined from fits to the RDC-refined X-ray structure [8–10] under the assumption of uniform fluctuations of 10° of the *ϕ* angles [16]. The highly dynamic residues 12 and 40 were excluded, and three ubiquitin residues with positive *ϕ* angles, namely residues 46, 60 and 64, were included in the fits, using angles from an NMR-refined X-ray structure [17]. The Karplus curve coefficients ^3^*J*_HN,Hα_(*ϕ*)=8.754 cos^2^*ϕ*−1.222 cos*ϕ*+0.111 Hz, ^3^*J*_HN,Cβ_(*ϕ*)=3.693 cos^2^*ϕ*−0.514 cos*ϕ*+0.043 Hz, and ^3^*J*_HN,C′_(*ϕ*)=4.516 cos^2^*ϕ*−1.166 cos*ϕ*−0.038 Hz were obtained.

It has been shown that the values for ^3^*J*_HN,Hα_, ^3^*J*_HN,Cβ_ and ^3^*J*_HN,C′_ of GB3 can be predicted somewhat better if density functional theory (DFT) calculations are performed on the structure of the RDC refined X-ray structure (PDB 2OED) than from simple parametrization of the experimental data by Karplus curves [18]. This demonstrates that some discrepancy between the Karplus curves and experimental data is caused by hydrogen bonding, substituent and electrostatic effects rather than fluctuation of the dihedral angles. These errors are very small for ^3^*J*_HN,Hα_ and ^3^*J*_HN,C′_ and are clearly dominated by the experimental errors (which are used for the width of the flat bottom CYANA potential). The situation is somewhat different for ^3^*J*_HN,Cβ_. Here, the error due to the substituent effect is ca. 0.08 Hz. Thus, the errors for ^3^*J*_HN,Cβ_ were set to the propagated errors obtained from the individual random experimental errors plus a uniform error of 0.08 Hz. This increases the overall error from 0.07 Hz to 0.11 Hz.

^3^*J*_Hα,Hβ2_ and ^3^*J*_Hα,Hβ3_ values were taken from reference [12], where a 3D HBCBCA type experiment provides two independent values allowing for a cross-check. A systematic error arises from the errors in the extracted peak positions caused by ^1^H^α^ transverse relaxation during the S^3^CT element and is estimated to be 1 Hz (see Fig. 4 in the Supplemental information in Ref. [12]). The systematic and individual random errors were propagated into an overall error. All couplings are in agreement with our previously calculated ensemble [2,3] except for residues 8 and 52. Residue 8 seems to undergo averaging as indicated by the two nearly identical values of ^3^*J*_Hα,Hβ2_ and ^3^*J*_Hα,Hβ3_ close to 7 Hz. These values are used here since a potentially wrong stereoassignment would not have an impact. The couplings of residue 52 are also in disagreement with the data set in reference [19], the X-ray structure [8], and our eNOE-based stereospecific assignment [20], all of which suggest a single rotamer state. On the other hand, the ^3^*J*_C’,Cγ_ and ^3^*J*_N,Cγ_ couplings for residue 52 in reference [2] appear to be slightly averaged over at least two rotamer states. Due to these inconsistencies, the ^3^*J*_Hα,Hβ2_ and ^3^*J*_Hα,Hβ3_ couplings of residue 52 are not used here. The substituent-effect-corrected Karplus parametrization (A, B, C)=(7.23, −1.37, 2.40) is used as proposed for Arg, Asx, Glx, His, Leu, Lys, Met, Phe, Pro, Trp and Tyr in Ref. [21].

We determined ^3^*J*_C’,Cγ_ and ^3^*J*_N,Cγ_ couplings for aromatic residues using the pulse sequences proposed in Ref. [22]. ^3^*J*_C’,Cγ(1/2)_ and ^3^*J*_N,Cγ(1/2)_ couplings for Val, Ile and Thr residues were taken from Ref. [23]. The Karplus coefficients for the aromatic residues were taken from Ref. [21] proposing (A, B, C) to be (2.31, −0.87, 0.49/1.29, −0.49, 0.34), and for the methyl bearing residues (2.76, −0.67, 0.19/2.01, 0.21, −0.12) for Thr and (3.42, −0.59, 0.17/2.64, 0.26, −0.22) for Val and Ile as proposed in Ref. [23].

### 2.4 Torsion angle restraints from C^α^ chemical shifts

Restraints for the *ϕ* and *ψ* backbone torsion angles were generated from ^13^C^α^ chemical shifts with CYANA [24,25]. The allowed ranges were chosen conservatively [26] and are either −200° to −80° for *ϕ* and 40–220° for *ψ* if the ^13^C^α^ secondary chemical shift was larger than 2 ppm, −120° to –20° for *ϕ* and –100° to 0° for *ψ* if the ^13^C^α^ secondary chemical shift was less than –1.5 ppm, or −120° to 80° for *ϕ* and −100° to 60° for *ψ* if the ^13^C^α^ secondary chemical shift was between 1.5 and 2 ppm. No torsion angle restraints were generated if the ^13^C^α^ secondary chemical shift was between –1.5 and +1.5 ppm.

## Figures and Tables

**Table 1:** Compiled CYANA restraints from GB3.

Type				*#*		
eNOEs	upper/lower distance limit	total		984		
		bidirectional	total		355	
			involving pseudo-atom^a^			31
			else			324
		unidirectional	total		568	
			involving pseudo-atom^a^			69
			else			499
NOEs	upper distance limit	aromatics^b^			61	
scalar couplings	total			225		
	backbone	total			147	
		^3^*J*_HN,Hα_				49
		^3^*J*_HN,Cβ_				49
		^3^*J*_HN,C′_				49
	backbone-side chain	total			78	
		^3^*J*_Hα,Hβ2/3_				24
		^3^*J*_N,Cγ(1/2)_				27
		^3^*J*_C’,Cγ(1/2)_				27
RDCs	total			1477		
	backbone	^1^*D*_HN,N_	total		372	
			medium 1			50
			medium 2			54
			medium 3			54
			medium 4			52
			medium 5			54
			medium 6			54
			medium 7			54
		^1^*D*_Cα,Hα_	total		387	
			medium 1			43
			medium 2			50
			medium 3			49
			medium 4			47
			medium 5			50
			medium 6			49
			medium 7			50
			medium 8			49
		^1^*D*_Cα,C’_	total		320	
			medium 2			54
			medium 3			54
			medium 4			50
			medium 5			54
			medium 6			54
			medium 7			54
		^1^*D*_HN,C’_	total		256	
			medium 2			52
			medium 3			51
			medium 4			47
			medium 5			54
			medium 6			52
	backbone-side chain	^2^*D*_Cβ,Hα_	medium 1		13	13
	side chain	^1^*D*_Cβ,Hβ(2/3)_	total		54	
			medium 1			38
			medium 8			16
		^1^*D*_Hβ2,Hβ3_	medium 1		11	11
		^1^*D*_Cm,Hm3_	total		64	
			medium 1			32
			medium 8			32
Angular restraints	total			52		
	phi					26
	psi					26

a Pseudo-methylene atom or pseudo-atom for both methyl groups in Val and Leu.

b NOEs involving aromatic groups, set to <8 Å.
